# Association between type 1 diabetes mellitus and dental caries in children and adolescents: a meta-analysis and Mendelian randomization study

**DOI:** 10.3389/fendo.2026.1929819

**Published:** 2026-09-07

**Authors:** Keyi Zhu, Fei Gao, Junzhu Yue, Miaoxin Yang, Jingyang Wu, Ruyi Zhao, Duo Li

**Affiliations:** 1 Dept of Stomatology, The Affiliated People’s Hospital of Ningbo University, Ningbo University, Ningbo, Zhejiang, China; 2 Health Science Center, Ningbo University, Ningbo, Zhejiang, China

**Keywords:** adolescents, children, dental caries, Mendelian randomization analysis, meta-analysis, type 1 diabetes mellitus

## Abstract

**Introduction:**

This study aimed to examine the relationship between type 1 diabetes mellitus (T1DM) and dental caries among children and adolescents through a meta-analysis, and to assess the potential causal link between these conditions using Mendelian randomization (MR) techniques.

**Methods:**

A comprehensive literature review was carried out to identify relevant studies, and meta-analyses were conducted utilizing Stata statistical software. For the MR analyses, summary-level data from genome-wide association studies (GWAS) regarding T1DM and dental caries in childhood and adolescence were gathered.

**Results:**

The meta-analysis indicated that the decayed, missing, and filled teeth (DMFT) index for permanent teeth was notably higher in children with T1DM compared to the control group, yielding a pooled standardized mean difference (SMD) of 0.19 (95% CI: 0.06–0.31; I^2^ = 26.6%). However, the MR analysis did not reveal a significant causal relationship between T1DM and the prevalence of dental caries in children and adolescents (OR = 0.98, 95% CI: 0.94–1.02, P = 0.308).

**Discussion:**

While children and adolescents with T1DM are at an elevated risk for dental caries, no statistically significant causal link was observed.

**Systematic review registration:**

https://www.crd.york.ac.uk/PROSPERO/view/177851 identifier CRD2020177851.

## 1 Introduction

Type 1 diabetes mellitus (T1DM) is a metabolic condition characterized by the complete absence of insulin production due to autoimmune destruction of pancreatic β-cells (1). This condition predominantly affects children and adolescents, with approximately 75% of cases diagnosed before the age of 18 years (2). t is estimated that 1.9 million individuals under the age of 20 years worldwide are living with T1DM, with more than 100,000 new cases diagnosed annually (3). The increasing incidence of T1DM highlights its importance as a critical public health concern requiring immediate attention, particularly in the pediatric population.

Extensive clinical research indicates that persistent hyperglycemia in T1DM can induce low-grade systemic inflammation, impair peripheral vascular microcirculation (4), and disrupt salivary gland function as well as the balance of oral microbial communities (5). Through multiple pathological mechanisms, T1DM exerts long-term adverse effects on oral health, increasing the risk of dental caries, gingivitis, plaque accumulation, xerostomia, and oral mucosal lesions (6).

Glycemic control status is a key determinant of oral infection risk in pediatric patients with diabetes mellitus, and its role can be systematically elucidated through a tripartite analytical framework—the “glycemic control–oral microecology–infection phenotype” axis model. Accumulating evidence consistently demonstrates that poor glycemic control is significantly associated with an increased prevalence and greater severity of dental caries, periodontitis, and candidal infections in this population (7).

Dental caries, recognized as the most prevalent chronic infectious disease among children worldwide, results from complex interactions among acid-producing microorganisms, carbohydrate consumption, and host factors (8). Chronic hyperglycemia associated with T1DM is considered a significant contributor to the initiation and progression of caries (5). Recent epidemiological studies investigating caries prevalence in children with T1DM have consistently shown that subgroups with poor glycemic control, as stratified by HbA1c levels, exhibit a significantly higher caries burden, whereas studies without such stratification are more likely to report null findings. This observation is consistent with the conclusions of Popoviciu et al., who emphasized that HbA1c-based stratification is associated with substantial differences in metabolic status, diabetes-related complications, cumulative comorbidity burden, and estimated complication risk (9).

Although several studies have identified various susceptibility factors associated with caries in children with T1DM, the relationship between T1DM and the prevalence and severity of dental caries remains unclear (10). Many studies have reported a markedly higher prevalence and severity of caries among children with T1DM, which may be explained by biological mechanisms (11). Prolonged hyperglycemia may impair oral microcirculation, leading to reduced salivary flow and decreased buffering capacity (12, 13). Saliva plays an essential role in neutralizing oral acidity, promoting enamel remineralization, and inhibiting the growth of cariogenic bacteria; therefore, salivary gland dysfunction may theoretically increase the risk of caries (14, 15). Furthermore, elevated glucose levels can significantly alter the composition and abundance of key cariogenic bacteria, including Streptococcus mutans and Lactobacillus species, potentially accelerating caries progression (16, 17).

Conversely, some studies have suggested that the incidence of dental caries in children with T1DM is not significantly increased and may even be lower than that observed in healthy controls (5). This finding may be attributed to several interrelated factors. Strict dietary management in patients with T1DM limits the intake of fermentable carbohydrates, thereby reducing substrate availability for acidogenic oral bacteria and decreasing caries risk (18). In addition, chronic hyperglycemia may enhance salivary antimicrobial components, such as antimicrobial peptides and immunoglobulins, thereby strengthening innate oral immunity and limiting the proliferation of cariogenic microorganisms (19). Long-term standardized follow-up care for pediatric patients with T1DM typically includes continuous oral health education and hygiene practices, which may effectively mitigate the cariogenic effects of hyperglycemia (5).

Factors such as glycemic control (HbA1c), disease duration, dietary adherence, oral health behaviors, and socioeconomic status may simultaneously influence both T1DM progression and caries development; however, traditional study designs are insufficient to adequately adjust for these complex confounding factors (20).

This study aims to conduct a systematic review and meta-analysis to synthesize recent observational data and quantitatively evaluate differences in caries prevalence and severity between children and adolescents with T1DM and healthy controls, thereby elucidating the strength of their clinical association. Furthermore, by employing genetic variants as instrumental variables, this study will investigate the potential causal relationship between T1DM and childhood caries from a genetic perspective, thereby improving the reliability and scientific rigor of causal inference and providing robust evidence-based medical support for future mechanistic investigations and the development of oral health intervention strategies for children with T1DM. The novelty of this study lies in the construction of a dual-layered evidence chain integrating clinical observations with genetic causal inference, combined with a methodological approach specifically targeting a well-defined pediatric population. This design aims to provide a more robust and comprehensive scientific foundation for understanding the relationship between T1DM and childhood dental caries, thereby offering high-quality evidence-based medical support for future mechanistic investigations and the development of oral health intervention strategies in children with T1DM.

## 2 Materials and methods

### 2.1 Meta-analysis

#### 2.1.1 Protocol and registration

The protocol for this systematic review and meta-analysis was registered with the International Prospective Register of Systematic Reviews (PROSPERO) under the registration number CRD2020177851. The complete manuscript was developed in accordance with the guidelines outlined in the Preferred Reporting Items for Systematic Reviews and Meta-Analysis Protocols (PRISMA-P) (21).

#### 2.1.2 Search strategy

A comprehensive computerized literature search was conducted in five electronic databases: the Cochrane Library, Embase, PubMed, Scopus, and Web of Science. The search included studies published up to January 11, 2026, without restrictions on language, geographical location, or publication year. The search strategy incorporated a combination of Medical Subject Headings (MeSH) terms and relevant synonyms and related terms, including “dental caries,” “children,” and “diabetes mellitus.” Furthermore, the reference lists of all included studies and relevant reviews were manually screened to identify additional eligible studies. Grey literature was also searched using the Google Scholar search engine. When multiple studies were derived from the same baseline population, only the study with the most recent data and the most comprehensive baseline information was included in the analysis.

#### 2.1.3 Inclusion and exclusion criteria

The inclusion criteria were defined as follows: (1) study design: cross-sectional studies or baseline data from longitudinal cohort studies; (2) study population: children under 12 years of age and adolescents aged 12 to 18 years; (3) diagnosis of dental caries: studies that strictly followed the World Health Organization (WHO) criteria and used both deciduous and permanent dentition assessment systems, with the presence of caries defined as dmft/DMFT/DMFS/dmfs > 0; (4) all participants were clinically diagnosed with T1DM. (dmft: decayed, missing, filled teeth for primary dentition; DMFT: Decayed, Missing, Filled Teeth for permanent dentition; DMFS: Decayed, Missing, Filled Surfaces for permanent dentition; dmfs: decayed, missing, filled surfaces for primary dentition) The exclusion criteria were as follows: (1) studies that were not original research, including reviews, commentaries, or case reports; however, studies for which citation information could be fully traced back to the original research were reconsidered on an individual basis; (2) studies focusing on adult populations; (3) studies lacking essential data or from which valid pooled effect estimates could not be calculated; (4) duplicate publications, overlapping samples, or redundant data.

#### 2.1.4 Quality assessment and data extraction

Two independent researchers conducted the preliminary literature screening, standardized the data extraction process, and assessed the risk of bias. Any disagreements between the two reviewers that could not be resolved through discussion were referred to a third senior researcher for adjudication.

The methodological quality of the cross-sectional studies was assessed using the standardized evaluation scale recommended by the Agency for Healthcare Research and Quality (AHRQ), which consists of 11 items. Each item rated as “yes” received 1 point, whereas items rated as “no” or “unclear” received 0 points, resulting in a maximum total score of 11 points. Studies with scores ranging from 4 to 7 points were categorized as moderate quality, whereas those with scores ranging from 8 to 11 points were classified as high quality. To ensure the reliability of the evidence-based conclusions, only high-quality studies with scores of 8 or higher, indicating a low risk of bias, were included in the subsequent statistical analyses. Studies with scores below 8, indicating a moderate to high risk of bias, were excluded.

#### 2.1.5 Data extraction specification

Studies that met the eligibility criteria were identified, and key research data were extracted according to the predefined inclusion and exclusion criteria. When multiple publications originated from the same study population, only the dataset with the most comprehensive sample information and the most recent updates was selected. The standardized data extraction included the following variables: the first author’s name, year of publication, country of study, total sample size, gender distribution, baseline age, glycemic control level among individuals with T1DM, and prevalence of dental caries.

#### 2.1.6 Statistical analysis

All statistical analyses were performed using Stata version 16.0. The pooled effect sizes for caries outcomes from the included studies were calculated using the DerSimonian-Laird random-effects model combined with the generic inverse variance method. Statistical heterogeneity among studies was assessed using the I² statistic. Sensitivity analyses were performed by sequentially excluding individual studies to evaluate their influence on the overall pooled effect size and to assess the robustness of the findings.

#### 2.1.7 Meta-regression analysis

Meta-regression analysis was conducted to investigate potential sources of heterogeneity among studies. Univariate meta-regression analyses were performed for each covariate of interest, and variables with a P-value < 0.05 in the final model were considered statistically significant.

### 2.2 Mendelian randomization analysis

**Figure 1:**
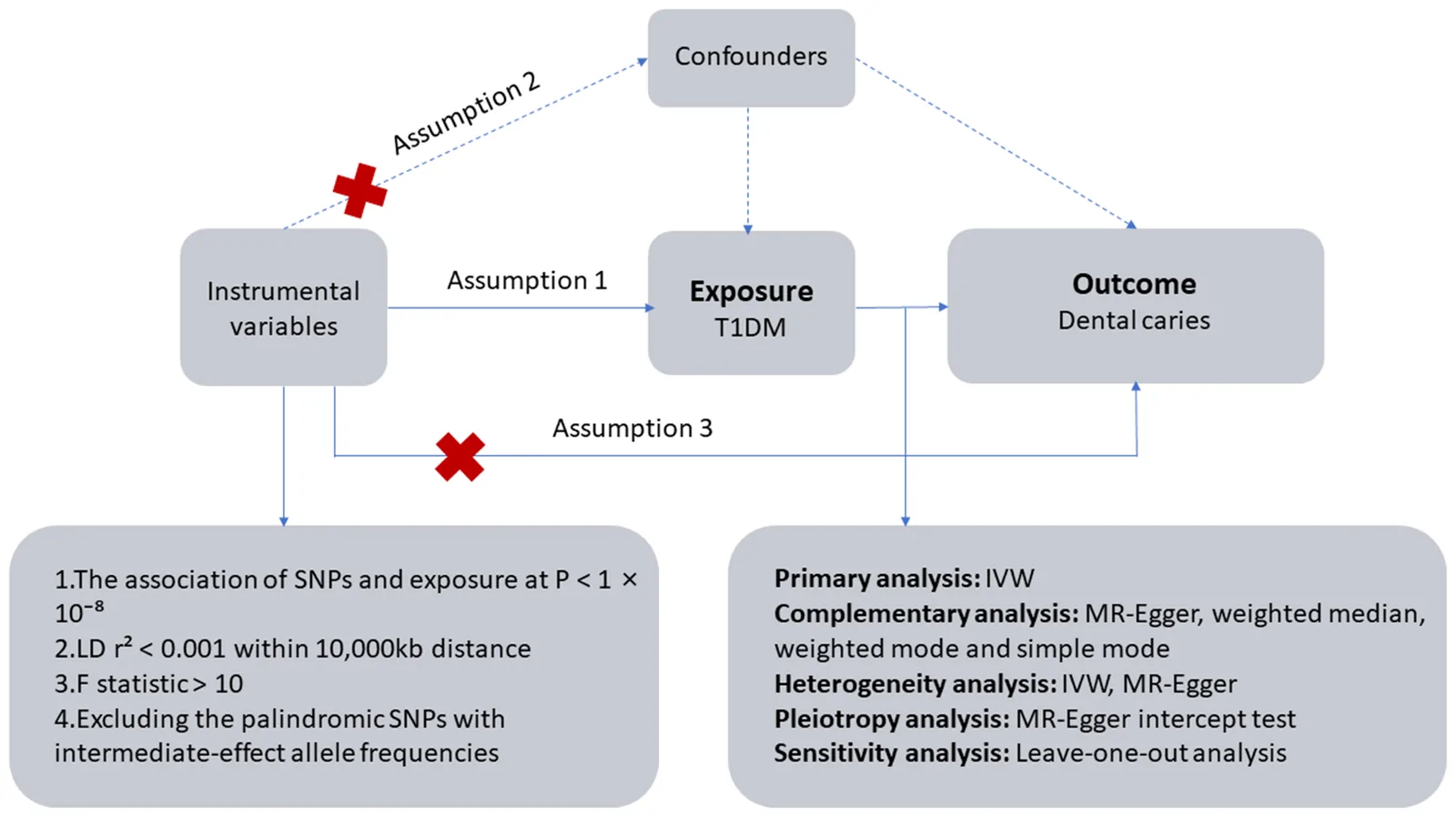
Study design. Assumption 1: There is a significant association between the exposure and genetic variants. Assumption 2: The instrumental variables are independent of confounders. Assumption 3: The instrumental variables affect the outcome only through the exposure and are independent of the outcome otherwise. T1DM: Type 1 diabetes mellitus; IVW: Inverse variance weighting; LD: Linkage disequilibrium; MR: Mendelian randomization; SNP: Single nucleotide polymorphism.

Two-sample Mendelian randomization (MR) analyses were conducted using genome-wide association study (GWAS) summary statistics to investigate the potential causal relationship between T1DM and both deciduous and permanent dental caries in children and adolescents. This study strictly adhered to the three core assumptions of MR: (1) the instrumental variables (IVs) must be strongly associated with the exposure;(2) the IVs must be independent of potential confounding factors; and (3) the IVs must influence the outcome exclusively through the exposure pathway, without direct effects or confounding mechanisms (22). The entire analytical process followed the Strengthening the Reporting of Observational Studies in Epidemiology using Mendelian Randomization (STROBE-MR) guidelines. The overall study design is illustrated in **Figure 1**.

#### 2.2.1 Data sources

##### 2.2.1.1 GWAS summary data for childhood caries

Genetic association data for both deciduous and permanent dental caries were obtained from the Avon Longitudinal Study of Parents and Children (ALSPAC) (23), which included individuals of European ancestry aged 3.0 to 18.0 years. Caries outcomes were assessed through clinical oral examinations or standardized questionnaires. Among participants of European ancestry, the deciduous caries dataset included 9,502 individuals (3,631 cases and 5,871 controls), whereas the permanent caries dataset included 9,781 individuals (3,193 cases and 6,588 controls). The data are available at: https://data.bris.ac.uk/datasets/pkqcnil6e9ju2nyreblt3mvwf/summary_statistics/.

##### 2.2.1.2 GWAS summary data for T1DM

Genetic summary data for T1DM were obtained from a comprehensive study involving adult populations of European ancestry. Due to the lack of a publicly available large-scale GWAS dataset for childhood T1DM, adult summary-level data were used as genetic instrument data for the exposure in this study. The dataset included 520,580 individuals, comprising 18,942 cases and 501,638 controls. These data are publicly available at: https://opengwas.io/datasets/ebi-a-GCST90014023.

All analytical data used in this study were obtained from publicly available resources that complied with ethical standards and had received approval from the relevant ethics committees and institutional review boards. Exposure and outcome data were obtained from independent GWAS consortia, thereby minimizing potential bias caused by sample overlap. Both datasets consisted of individuals of European ancestry, ensuring consistency in genetic background.

#### 2.2.2 Instrumental variable selection

Single nucleotide polymorphisms (SNPs) that showed robust associations with T1DM exposure were identified using a genome-wide significance threshold of P < 1×10^-^^8^. To account for linkage disequilibrium (LD), clumping was performed using a window size of 10,000 kb and an r² threshold of < 0.001. The F-statistic was calculated for each SNP, and SNPs with F < 10, indicating weak instrument strength, were excluded from the analysis. In addition, palindromic SNPs with intermediate allele frequencies were removed. The remaining eligible SNPs were subsequently used as genetic instrumental variables in this study (24).

#### 2.2.3 Primary Mendelian randomization analysis

A two-sample MR design was employed to evaluate the potential causal effect of T1DM on the prevalence of dental caries in children and adolescents. All primary and sensitivity analyses were performed using R software (version 4.5.2.0) with the Two Sample MR package. Four established MR methods were applied, including fixed-effect inverse variance weighted (IVW-FE), random-effect inverse variance weighted (IVW-RE), weighted median (WM), and maximum likelihood (ML) methods. The fixed-effect inverse variance weighted model (IVW-FE), which is the most commonly used method in this research field, was selected as the primary analytical model. To account for multiple testing, the false discovery rate (FDR) correction was applied, and a corrected P-value < 0.05 was considered statistically significant.

#### 2.2.4 Sensitivity analysis

Comprehensive sensitivity analyses were conducted to evaluate the robustness of the causal estimates. As the selected SNPs were derived from multi-center GWAS datasets, Cochran’s Q statistic was used to assess heterogeneity. Horizontal pleiotropy was evaluated using the MR-Egger intercept test and the Mendelian Randomization Pleiotropy RESidual Sum and Outlier (MR-PRESSO) method to identify potential outlier SNPs. Additionally, a leave-one-out analysis was performed to determine whether any individual genetic variant had a substantial influence on the overall causal estimates.

## 3 Results

### 3.1 Meta-analysis results

#### 3.1.1 Literature search and quality assessment results

**Figure 2:**
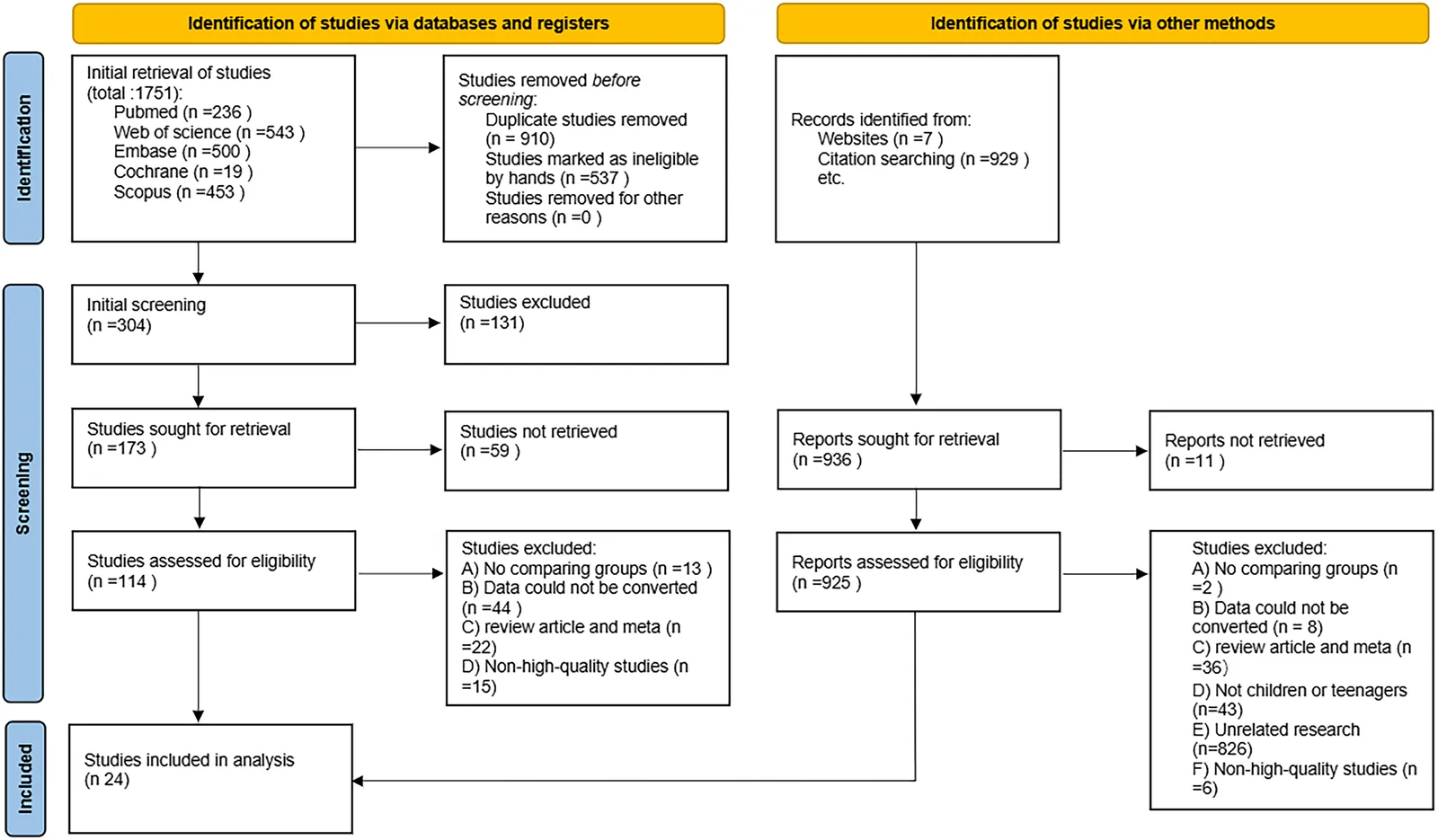
Flowchart of the literature search process.

**Figure 3:**
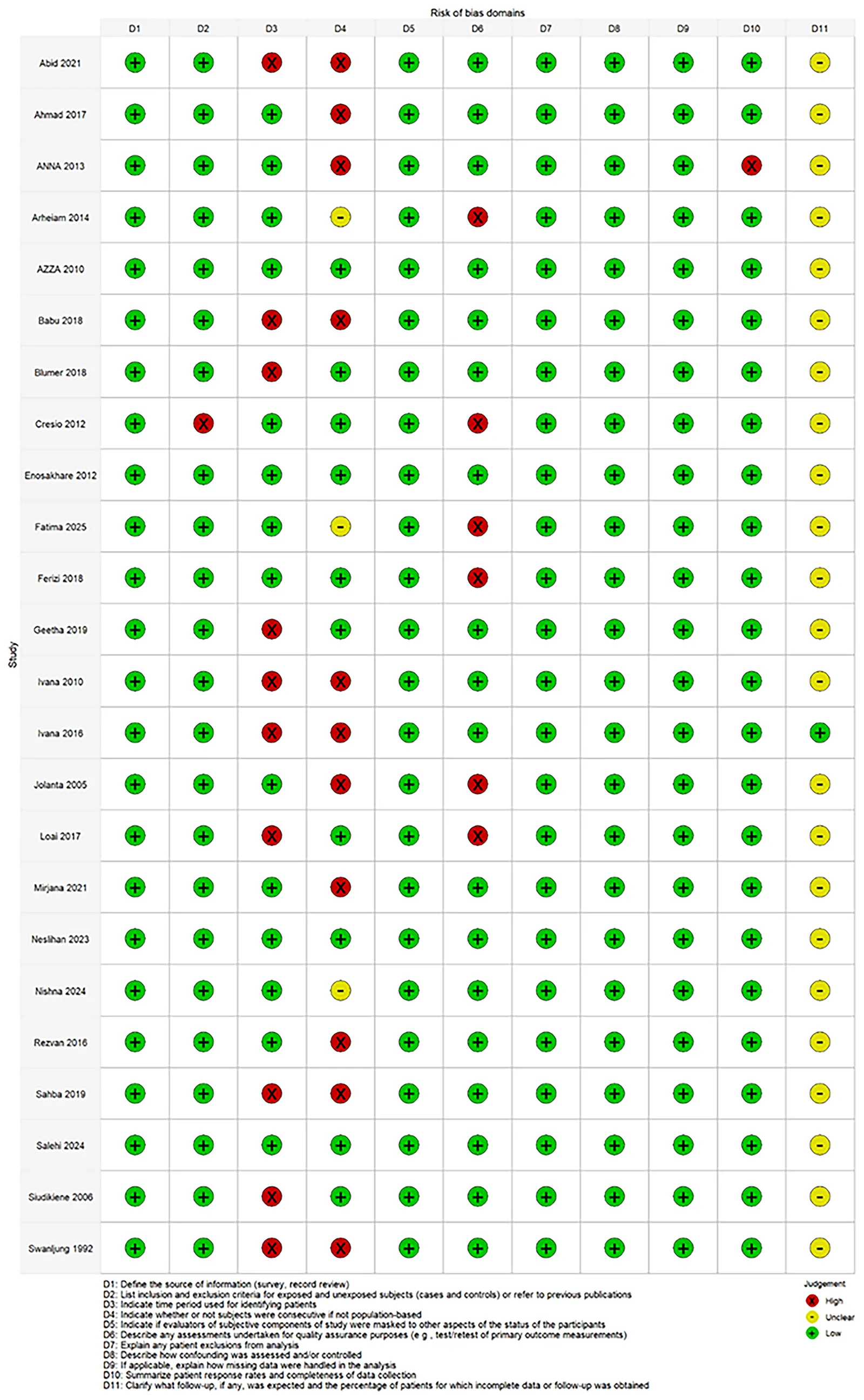
AHRQ quality assessment of the 24 cross-sectional studies.

**Figure 4:**
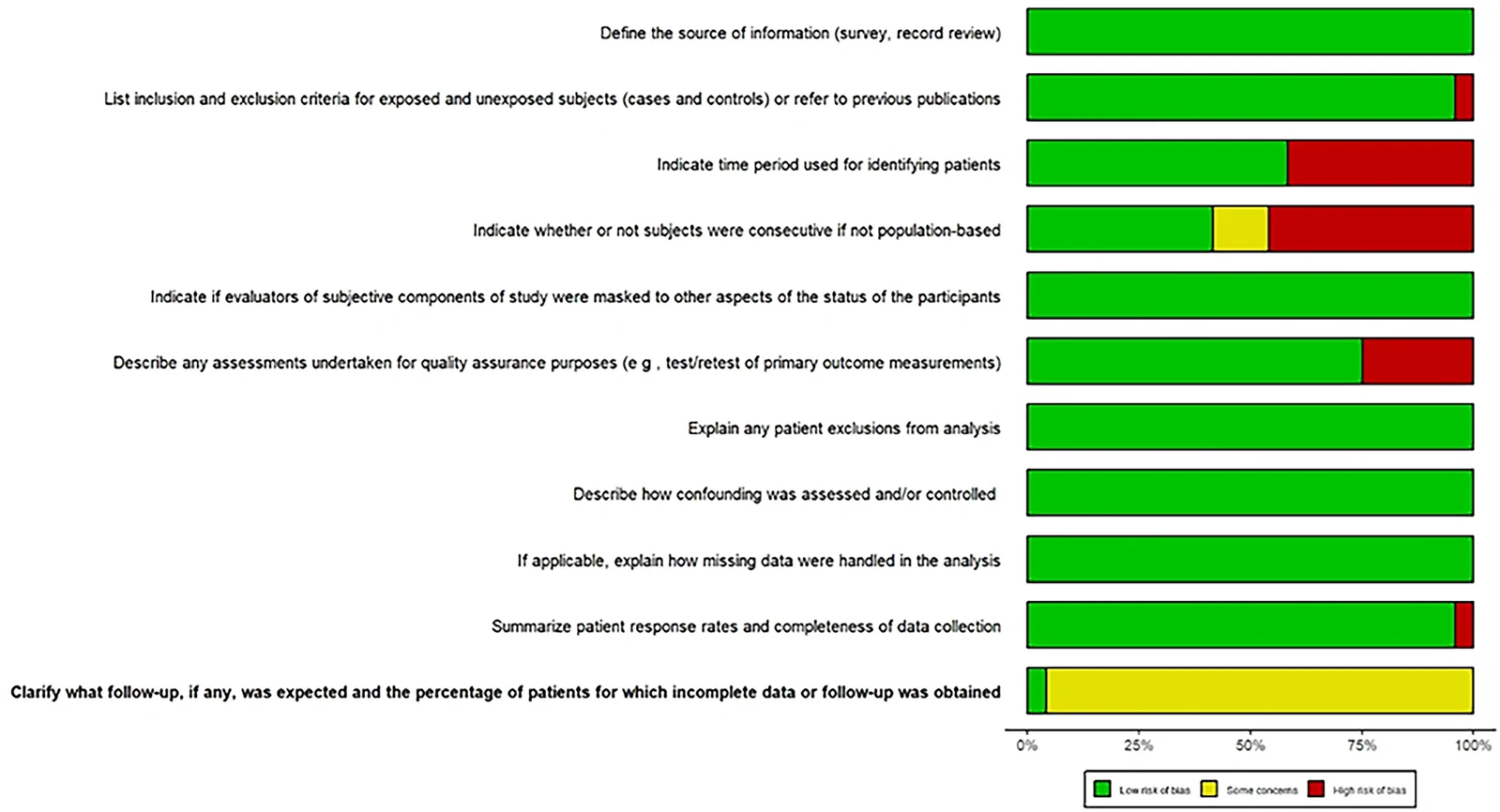
Summary of the overall risk of bias for the 24 studies.

The preliminary search identified 1,751 relevant records. Following a stepwise screening process, 1,706 articles were excluded due to reasons including duplicate publications, lack of odds ratios (ORs) and 95% confidence intervals (CIs), non-clinical study designs, or studies involving non-human subjects (**Figure 2**). The remaining studies were assessed for risk of bias using the AHRQ tool, and 21 studies were classified as having a high risk of bias. To evaluate the impact of excluding moderate-quality studies, sensitivity analyses were performed by incorporating studies with an AHRQ score < 8. The pooled effect estimates remained stable and consistent with the primary analysis, supporting the robustness of the findings. However, inclusion of these studies resulted in substantially increased heterogeneity, which may limit the interpretation of the overall effect. Therefore, the primary analysis was restricted to high-quality studies (AHRQ ≥ 8) to ensure more reliable and interpretable results. Ultimately, 24 high-quality studies were included in the analysis (13, 18, 25–46). A detailed summary of the risk of bias assessment for the included studies is provided in the **Supplementary Materials (S1)**, **Figures 3** and **4**. The basic characteristics of the included studies are presented in **Table 1**.

**Table 1:** Characteristics of included studies.

Authors,years	Country	Gender(M%)	age (years)	Mean HbA1c	Total sample size	Dental caries measurement	Sample size of type 1 diabetes	Mean (SD) DMFT/dmft/DMFS/dmfs
Abid et al,2021	Saudi Arabia	46%	6-12	/	209	DFT/dft	69	1.62(0.65)/3.32(0.78)
Ahmad et al,2017	China	50%	4-17	7.51 ± 1.28%	64	DMFT/dmft	32	1.69(1.75)/1.09(2.43)
ANNA et al,2013	Poland	50%	7-17	DP permanent dentition:7.51 ± 2.04%; DM mixed dentition:7.51 ± 1.46%	120	DMFT/DMFT(DM) dmft(DM)	60	13.6(7.6)/16.9(9.3) 5.4(6.5)
Arheiam et al,2014	Libya	64%	10-15	/	140	DMFT	70	1.19(1.74)
AZZA et al,2011	Belgium	56%	3-16	7.85 ± 0.82	102	DMFS/dmfs/DMFT/dmft	52	5.61(5.97)/3.89(3.81)/3.84(3.89)/2.86(2.52)
Babu et al,2018	India	52.50%	6–18	/	160	DMFT/Deft	80	1.26(2.49)/0.44(1.28)
Blumer et al,2018	Israel	54.17%	2-11	8.0 ± 1.3%	54	DMFT/dmft	24	2.72(3.3)/2.4(2.9)
Cresio et al,2012	Brazil	56.90%	6-18	/	102	DMFT/deft	51	1.94(2.84)/0.64(1.24)
Enosakhare et al,2012	Kuwait	47.00%	12-15	controlled 7%;uncontrolled:10%	106	*DMFT/*DFS	53	6.4(4.7)/7.3(6.5)
Fatima et al,2025	India	53%	3-17	/	200	average DMFT/dmft index	100	6.1(3.26)
Ferizi et al,2018	Kosovo	49%	10-15	/	160	DMFT	80	6.56(3.56)
Geetha et al,2019	India	41.70%	10-15	/	350	DMFT/dmft	175	0.7(0.45)/0.26(0.05)
Ivana et al,2010	Brazil	47.10%	14-19	GHb:9.8%	102	DMF	51	3.3(3.7)
Ivana et al,2016	Brazil	43.75%	14-19	9.8 ± 2.4%	64	DMFT	32	4(0.7)
Jolanta et al,2005	Lithuania	/	10-15	/	140	DMFS	70	23.07(14.38)
Loai et al,2017	Syria	100%	2-15	8.92 ± 2.72%	135	DMFT	95	5.46(1.82)
Mirjana et al,2021	Montenegro	55.17%	10-15	9.9 ± 1.7%	177	DMFT	87	4.3(1.79)
Neslihan et al,2023	Turkey	49.20%	3-15	9.3 ± 2.5%	122	dmft/DMFT	65	6.9(3.9)
Nishna et al,2024	India	49.50%	5-14	/	200	DMFT	100	2.28(2.44)
Rezvan et al,2016	Iran	/	5-18	8.54 ± 1.62%	160	DMFT/dmft	80	3.78(3.24)/2.52(3.29)
Sahba et al,2019	Iran	43%	9-14	/	200	DMFT	100	2.6(1.25)
Salehi et al,2024	Iran	57.60%	2-5	/	66	dmft	33	5.55(1.1)
Siudikiene et al,2006	Lithuania	47%	10-15	Good control:7.60 ± 0.95;poor control:10.35 ± 0.99	136	DMFS	68	23.03(14.54)
Swanljung et al,1992	Finland	45.90%	12-18	10.9 ± 2.2%	170	DMFS/DMF	85	5.8(5)/4.3(3.1)

#### 3.1.2 Analysis of DMFT and DMFS index

**Figure 5:**
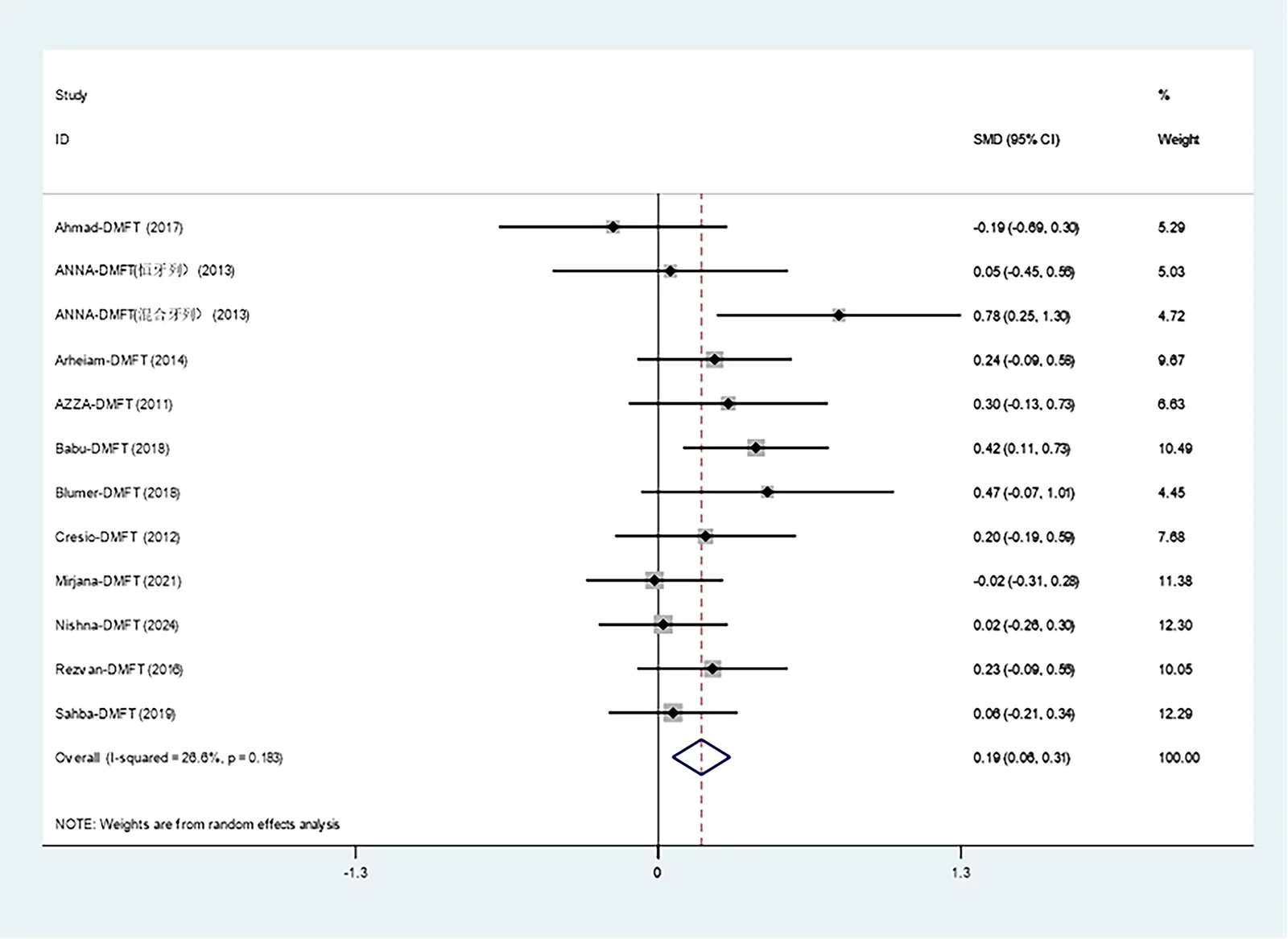
Forest plot of pooled effect sizes (DMFT).

The pooled analysis of 12 studies evaluating DMFT outcomes using a random-effects model demonstrated that the DMFT index was significantly higher in the T1DM group than in the control group (standardized mean difference [SMD] = 0.19, 95% CI: 0.06–0.31), with low heterogeneity among studies (I² = 28.6%, P = 0.183). These findings indicate that permanent tooth caries status is significantly worse in children with T1DM compared with healthy controls (**Figure 5**).

In the time-based subgroup analysis, studies published before 2020 showed a pooled SMD of 0.24 (95% CI: 0.10–0.38), whereas studies published after 2020 showed a pooled SMD of 0.00 (95% CI: -0.20–0.21), suggesting that publication year may influence the pooled effect size.

In the sex-based subgroup analysis, the pooled SMD was 0.24 (95% CI: 0.09–0.38) for studies with a male predominance, 0.04 (95% CI: -0.15–0.24) for studies with a female predominance, 0.21 (95% CI: -0.36–0.77) for studies with balanced sex distributions, and 0.23 (95% CI: -0.09–0.56) for studies without available sex information. These findings suggest that sex composition may contribute to between-study heterogeneity.

In the HbA1c-stratified subgroup analysis, the pooled SMD was 0.23 (95% CI: -0.16 to 0.62) for the < 8% group and 0.17 (95% CI: -0.08 to 0.41) for the ≥ 8% group, suggesting that glycemic control status does not substantially modify the overall effect.

In the disease duration-stratified subgroup analysis, the pooled SMD was 0.42 (95% CI: 0.17 to 0.88) for studies including patients with a disease duration of less than 1 month and 0.11 (95% CI: -0.04 to 0.27) for studies including patients with a disease duration of 1 month or longer, suggesting that disease duration may partially account for between-study heterogeneity.

In the confounder adjustment-stratified subgroup analysis, the pooled SMD was 0.09 (95% CI: -0.09 to 0.27) for studies that adjusted for confounding factors and 0.25 (95% CI: 0.09 to 0.41) for studies without adjustment for confounders, indicating that adjustment for confounding factors may partially explain the observed heterogeneity. No age-based subgroup analysis was conducted for DMFT.

The sensitivity analysis, performed by sequentially excluding individual studies, demonstrated that the overall effect size remained consistent with the original findings, and the 95% CI did not include zero, indicating the stability and reliability of the pooled results.

Univariate meta-regression analysis was performed to identify potential sources of heterogeneity among studies. Among all evaluated covariates, disease duration showed a marginally significant negative association with the effect size (β = -0.307, 95% CI: -0.644 to 0.030, P = 0.067), whereas publication year showed a borderline significant positive association (β = 0.233, 95% CI: -0.050 to 0.516, P = 0.096). However, no significant associations were observed for gender (P = 0.661), HbA1c level (P = 0.882), or adjustment for confounding factors (P = 0.237). These findings indicate that disease duration and publication year may represent potential sources of heterogeneity, while the other evaluated variables appear to have limited explanatory capacity.

To quantitatively assess publication bias, at least 10 studies are required. A funnel plot was generated to evaluate publication bias in the DMFT analysis, and the symmetrical distribution suggested a low risk of publication bias in this meta-analysis.

The pooled analysis of four studies evaluating DMFS outcomes using a random-effects model demonstrated no statistically significant difference in the DMFS index between the T1DM and control groups (SMD = 0.01, 95% CI: -0.36–0.38). Considerable heterogeneity was observed among the included studies (I² = 78.1%, P = 0.003). These findings indicate that the current evidence does not support a significant increase in DMFS among children with T1DM compared with healthy controls.

#### 3.1.3 Analysis of dmft index

A pooled analysis of eight studies evaluating dmft outcomes using a random-effects model showed no statistically significant difference in the dmft index between the T1DM and control groups (SMD = -0.12, 95% CI: -0.40–0.15), with moderate heterogeneity among the included studies (I² = 66.6%, P = 0.003). These findings suggest that the current evidence does not indicate a significantly poorer deciduous tooth caries status in children with T1DM compared with healthy controls.

In the time-based subgroup analysis, the pooled SMD was -0.21 (95% CI: -0.46 to 0.05) for studies published before 2020 and 0.46 (95% CI: -0.03 to 0.95) for studies published after 2020, indicating a potential difference in effect estimates according to publication year.

In the country-based subgroup analysis, the pooled SMD was -0.08 (95% CI: -0.52 to 0.37) for the European subgroup, -0.13 (95% CI: -0.47 to 0.21) for the Asian subgroup, and -0.33 (95% CI: -0.72 to 0.06) for the subgroup representing other countries. These findings suggest that the effect estimates were generally comparable across different geographical regions.

In the sex-based subgroup analysis, the pooled SMD was -0.02 (95% CI: -0.36 to 0.31) for studies with a male predominance, -0.05 (95% CI: -0.40 to 0.30) for studies with a balanced sex distribution, and -0.87 (95% CI: -1.40 to -0.35) for studies without available sex data, indicating that sex composition had a limited impact on the overall findings.

In the age-based subgroup analysis, the pooled SMD was -0.30 (95% CI: -0.50 to -0.09) for studies involving participants younger than 12 years and 0.46 (95% CI: 0.10 to 0.83) for studies involving participants aged 12 years or older. The effect directions differed substantially between these two groups, suggesting that age may be an important factor contributing to the heterogeneity observed among studies.

In the HbA1c-stratified subgroup analysis, the pooled SMD was -0.12 (95% CI: -0.40 to 0.16) for the < 8% group and -0.20 (95% CI: -1.52 to 1.12) for the ≥ 8% group, suggesting that glycemic control status may contribute to between-study heterogeneity.

In the disease duration-stratified subgroup analysis, the pooled SMD was 0.02 (95% CI: -0.42 to 0.46) for the < 1 year group and -0.55 (95% CI: -1.16 to 0.07) for the ≥ 1 year group. The presence of moderate heterogeneity within both subgroups suggests that disease duration may not substantially explain the observed variability among studies.

In the confounder adjustment-stratified subgroup analysis, the pooled SMD was 0.02 (95% CI: -0.40 to 0.43) for studies with adjustment for confounding factors and -0.20 (95% CI: -0.57 to 0.17) for studies without adjustment. The differences in within-group heterogeneity suggest that confounding control may partially account for the observed variability among studies.

Sensitivity analysis using the leave-one-out method demonstrated variations in the pooled effect estimates after sequential removal of individual studies. Exclusion of several studies resulted in pooled estimates with 95% CIs crossing zero, indicating limited stability of the primary findings.

Univariate meta-regression analysis was performed to identify potential sources of heterogeneity among studies. Among all evaluated covariates, age group showed a significant negative association with the effect size (β = -0.380, 95% CI: -0.645 to -0.114, P = 0.013), explaining 37.07% of the between-study variance. However, no significant associations were observed for gender (P = 0.718), publication year (P = 0.162), region (P = 0.854), HbA1c level (P = 0.872), adjustment for confounding factors (P = 0.770), or disease duration (P = 0.223). These findings indicate that age may be a key contributor to heterogeneity, whereas the remaining variables have limited explanatory capacity.

Given the limited number of included studies (n = 8), quantitative assessment of publication bias was not performed. However, qualitative evaluation of the funnel plot demonstrated slight asymmetry, indicating that the possibility of publication bias cannot be completely excluded.

Comprehensive results of the forest plot, sensitivity analysis, and funnel plots are provided in the **Supplementary Materials (S1)**.

### 3.2 Mendelian randomization analysis

#### 3.2.1 Causal effect of T1DM on permanent dental caries

A total of 67 SNPs associated with T1DM were identified as instrumental variables from GWAS data (F > 10, indicating the absence of weak instrument bias).

The primary analysis using the IVW method revealed no significant causal association between genetically predicted T1DM and permanent dental caries (odds ratio [OR] = 0.979, 95% CI: 0.939-1.020, P = 0.308). The results obtained from the other MR methods (IVW-RE, WM, ML) were consistent with these findings, with none showing statistical significance.

After adjustment for multiple testing, the causal association between T1DM and permanent dental caries remained statistically non-significant.

Sensitivity analyses demonstrated that the effect estimates and significance levels remained largely unchanged after excluding any individual SNP, indicating the robustness and stability of the results.

Heterogeneity assessments (IVW Q_p = 0.335; MR-Egger Q_p = 0.306) indicated no evidence of significant heterogeneity. The MR-Egger intercept test (P = 0.830) revealed no evidence of horizontal pleiotropy.

#### 3.2.2 Causal effect of T1DM on deciduous dental caries

A total of 67 SNPs associated with T1DM were identified as instrumental variables from GWAS data (F > 10, indicating the absence of weak instrument bias).

The primary analysis conducted using the IVW method revealed no significant causal association between genetically predicted T1DM and deciduous dental caries (OR = 0.982, 95% CI: 0.943–1.023, P = 0.416). Consistent results were observed across the other MR methods (IVW-RE, WM, ML), with none reaching statistical significance.

After adjustment for multiple comparisons, the causal association between T1DM and deciduous dental caries remained statistically non-significant.

Sensitivity analyses demonstrated that neither the effect estimates nor the significance levels were substantially altered after removal of any individual SNP, confirming the robustness and stability of the findings.

Heterogeneity assessments (IVW Q_p = 0.407; MR-Egger Q_p = 0.389) indicated no evidence of significant heterogeneity. The MR-Egger intercept test (P = 0.862) revealed no evidence of horizontal pleiotropy.

Figures summarizing the MR results are provided in the **Supplementary Materials (S2)**.

## 4 Discussion

This study employed a systematic review and MR analysis to comprehensively investigate the complex association between T1DM and childhood dental caries.

The impact of T1DM on caries demonstrates significant dentition-specific patterns. Among children with T1DM, the deft/dmft index for primary teeth did not show a significant increase, and some studies even reported lower values than those observed in healthy controls, whereas the DMFT/DMFS index for permanent teeth showed a marked increase or age-dependent progression (29). This discrepancy supports the hypothesis that the effects of T1DM on caries are influenced by dental tissue maturation and duration of exposure. Primary teeth, which have lower enamel mineralization, are subjected to strict sucrose restriction and enhanced oral care after diagnosis; these protective measures may counteract hyperglycemia-induced salivary dysfunction and reduce the risk of caries in primary dentition. In contrast, permanent teeth are exposed to systemic metabolic disturbances over a longer period. With prolonged disease duration, persistent hyperglycemia reduces salivary flow and buffering capacity while increasing glucose concentrations, thereby creating a cariogenic environment and accelerating enamel demineralization (47, 48). Furthermore, incomplete mineralization of permanent teeth during adolescence, combined with glycemic fluctuations, may place older children with T1DM at a substantially higher risk of caries than younger children (47, 49).

Temporal subgroup analysis showed that studies published before 2020 generally reported increased caries severity among children with T1DM, whereas more recent studies found no significant differences between groups, consistent with the findings of Wang et al. and Liu et al. (11, 50). This temporal shift may reflect improvements in comprehensive T1DM management, including intensified insulin therapy, continuous glucose monitoring, and targeted oral health education (48, 51). Strict dietary management, particularly reduced intake of refined carbohydrates, may have effectively attenuated the independent cariogenic effects of hyperglycemia (47).

Sex- and age-stratified analyses indicated that male children with T1DM may have a higher risk of permanent tooth decay under conditions of hyperglycemia and salivary dysfunction. In the subgroup of children younger than 12 years, those with T1DM exhibited significantly lower caries levels than healthy controls, whereas this association was reversed among those aged 12 years or older. This finding may be attributed to stricter dietary adherence among younger patients, in whom sucrose restriction may offset hyperglycemia-induced impairment of oral defense mechanisms (18). During adolescence, increased glycemic variability, tooth transition, and incomplete mineralization may collectively contribute to elevated caries risk (52). Geographic subgroup analysis revealed no significant differences in dmft across Europe, Asia, and other regions, suggesting that the influence of T1DM on primary dentition caries is predominantly driven by biological mechanisms, with limited effects from environmental, dietary, socioeconomic, or healthcare system-related factors.

HbA1c-stratified subgroup analysis revealed distinct roles of glycemic control in contributing to heterogeneity across different dentitions. In primary dentition, although pooled SMDs across HbA1c strata were close to zero and not statistically significant, the < 8% and ≥ 8% groups showed substantial differences in confidence interval width and the extent of zero crossing. This variability suggests that poorer glycemic control may correspond to greater between-study variation, indicating that glycemic control represents an important source of heterogeneity in primary dentition studies. In permanent dentition, subgroup results were highly consistent across HbA1c strata, with small effect sizes and overlapping confidence intervals, suggesting that glycemic control does not substantially modify the overall effect estimate and that the pooled effect remains stable across different metabolic states. The contribution of glycemic control to heterogeneity was greater in primary than in permanent dentition, highlighting the need for more rigorous glycemic stratification in meta-analyses and interventions involving children with primary dentition to minimize bias. In contrast, children with permanent dentition may require long-term comprehensive oral health management rather than reliance solely on short-term glycemic indicators (53).

Disease duration subgroup analysis in permanent dentition demonstrated that newly diagnosed patients (<1 month) had a significantly increased risk of caries; however, when disease duration extended to ≥1 month, the pooled effect size decreased substantially and lost statistical significance. This finding suggests that uncontrolled hyperglycemia, acute salivary dysfunction, and pre-existing metabolic disturbances during the early post-diagnosis period may directly accelerate demineralization of permanent teeth (49). Long-term intensive insulin therapy, dietary sucrose restriction, and improved oral hygiene practices may effectively reduce metabolism-related caries progression, making disease duration an important source of heterogeneity in permanent dentition studies (49, 54).

Nevertheless, existing dmft/DMFS studies demonstrate considerable heterogeneity, which is largely attributable to insufficient adjustment for confounding factors such as oral hygiene behaviors, family socioeconomic status, and parental oral health literacy (47, 55). Gunasekaran et al. reported that caries status in children with T1DM was more strongly associated with toothbrushing and flossing habits than with glycemic control alone (47). Similarly, ElBshari et al. suggested that the pathological effects of T1DM may be overshadowed by stronger environmental influences, including diet and obesity (47), which is consistent with our findings.

Meta-regression analysis identified age as a key determinant of heterogeneity among studies involving primary dentition, supporting the hypothesis that “protective behaviors counteract pathological risks.” Younger children with T1DM, who are subject to strict sucrose restriction and enhanced oral care, may exhibit dmft values even lower than those of healthy controls. The absence of significant regional differences further suggests that the effects of T1DM on primary dentition are primarily mediated by consistent biological mechanisms and early behavioral interventions, with limited geographical influence (49). For permanent dentition, meta-regression analysis indicated marginally significant effects of disease duration and publication year, suggesting that caries risk accumulation may depend more on long-term metabolic disturbances, including reduced salivary buffering capacity, incomplete mineralization, and progressive systemic inflammation, rather than immediate glycemic levels (49). Our findings suggest that the relationship between T1DM and childhood caries is more strongly influenced by acquired environmental factors and clinical management. Optimized preventive strategies for children with T1DM should emphasize topical fluoride application, individualized oral hygiene practices, strict glycemic control, and more frequent dental follow-up for early detection and intervention. Oral health education should be incorporated into diabetes self-management programs, covering key topics such as caries etiology, the relationship between glycemic control and oral health, appropriate brushing techniques, and fluoride toothpaste use. Collaboration between endocrinology and dentistry is essential to establish a “glycemic–oral care” synergy among families and effectively reduce caries risk.

Our MR analysis identified no causal relationship between genetically predicted T1DM and childhood caries, in contrast to the positive associations reported in adult cohorts by Tan et al. (2023) and Lim et al. (2025) (56, 57). This discrepancy may be attributable to age-dependent differences in the genetic architecture of T1DM. Childhood-onset T1DM is strongly influenced by high-risk HLA haplotypes (e.g., DR3-DQ2/DR4-DQ8), which are associated with high genetic risk scores and rapid disease progression (49, 58, 59), whereas adult-onset T1DM (including LADA) generally exhibits a lower genetic burden and greater contributions from environmental and epigenetic factors (60, 61). The application of adult GWAS-derived instrumental variables to pediatric cohorts may introduce cross-age applicability bias and weak instrument bias, as adult-associated non-HLA loci may exert weaker effects in childhood-onset disease and fail to capture childhood-specific HLA-driven genetic signals (62–64). Furthermore, MR associations observed in adults may reflect the cumulative effects of long-term hyperglycemia, including salivary alterations, microvascular complications, and immune dysfunction, representing potential accumulated reverse causation or bidirectional mechanisms. In contrast, children generally have shorter disease durations and fewer accumulated metabolic disturbances, which may explain why genetic susceptibility to T1DM does not significantly increase caries risk through direct biological pathways in pediatric populations (65–67).

The limitations of this study include the limited number of original dmft/DMFS studies included and incomplete reporting of important confounding factors, which may affect the stability of our conclusions. Additionally, the construction of instrumental variables using adult T1DM GWAS summary data while analyzing pediatric caries outcomes may introduce cross-age applicability bias and weak instrument bias, potentially representing a major reason for the null causal findings. Future studies should expand meta-analyses to include more ethnically diverse populations and increase the number of dmft- and DMFS-related studies to further validate the robustness of the findings. Large-scale, multi-ethnic GWAS studies specifically involving children and adolescents with T1DM are urgently needed to generate population-specific genetic data for elucidating underlying mechanisms. Moreover, larger pediatric longitudinal cohorts should be established to improve statistical power and ultimately facilitate the development of more precise early prevention strategies.

## 5 Conclusion

Meta-analysis revealed that children with T1DM had a significantly higher DMFT index than healthy controls, confirming a clinical association between T1DM and increased caries severity in permanent teeth. However, MR analysis did not support a causal genetic relationship between T1DM and dental caries, suggesting that the observed clinical association may not be directly driven by genetic factors. Given these limitations, further studies involving larger and more diverse cohorts are required to confirm the generalizability of our findings.

## Data Availability

The original contributions presented in the study are included in the article/**Supplementary Material**. Further inquiries can be directed to the corresponding author.
