# Supplementary material for: Association between type 1 diabetes mellitus and dental caries in children and adolescents: a meta-analysis and Mendelian randomization study
Source: Front Endocrinol (Lausanne). 2026 Sep 7;17:1929819. doi: 10.3389/fendo.2026.1929819 (PMC13590302; doi:10.3389/fendo.2026.1929819)
Supplement: Supplementary file 1 [file SupplementaryFile1.docx]

| Author Published Year | Define the source of information (survey, record review) | List inclusion and exclusion criteria for exposed and unexposed subjects (cases and controls) or refer to previous publications | Indicate time period used for identifying patients | Indicate whether or not subjects were consecutive if not population-based | Indicate if evaluators of subjective components of study were masked to other aspects of the status of the participants | Describe any assessments undertaken for quality assurance purposes (e.g., test/retest of primary outcome measurements) | Explain any patient exclusions from analysis | Describe how confounding was assessed and/or controlled. | If applicable, explain how missing data were handled in the analysis | Summarize patient response rates and completeness of data collection | Clarify what follow-up, if any, was expected and the percentage of patients for which incomplete data or follow-up was obtained | Total 'yes' |
| --- | --- | --- | --- | --- | --- | --- | --- | --- | --- | --- | --- | --- |
| Abid et al.(2021) | Yes | Yes | No | No | Yes | Yes | Yes | Yes | Yes | Yes | Unclear | 8 |
| Ahmad et al.(2017) | Yes | Yes | Yes | No | Yes | Yes | Yes | Yes | Yes | Yes | Unclear | 9 |
| ANNA et al.(2013) | Yes | Yes | Yes | No | Yes | Yes | Yes | Yes | Yes | No | Unclear | 8 |
| Arheiam et al.(2014) | Yes | Yes | Yes | Unclear | Yes | No | Yes | Yes | Yes | Yes | Unclear | 8 |
| AZZA et al.(2010) | Yes | Yes | Yes | Yes | Yes | Yes | Yes | Yes | Yes | Yes | Unclear | 10 |
| Babu et al.(2018) | Yes | Yes | No | No | Yes | Yes | Yes | Yes | Yes | Yes | Unclear | 8 |
| Blumer et al.(2018) | Yes | Yes | No | Yes | Yes | Yes | Yes | Yes | Yes | Yes | Unclear | 9 |
| Cresio et al.(2012) | Yes | No | Yes | Yes | Yes | No | Yes | Yes | Yes | Yes | Unclear | 8 |
| Enosakhare et al.(2012) | Yes | Yes | Yes | Yes | Yes | Yes | Yes | Yes | Yes | Yes | Unclear | 10 |
| Fatima et al.(2025) | Yes | Yes | Yes | Unclear | Yes | No | Yes | Yes | Yes | Yes | Unclear | 8 |
| Ferizi et al.(2018) | Yes | Yes | Yes | Yes | Yes | No | Yes | Yes | Yes | Yes | Unclear | 9 |
| Geetha et al.(2019) | Yes | Yes | No | Yes | Yes | Yes | Yes | Yes | Yes | Yes | Unclear | 9 |
| Ivana et al.(2010) | Yes | Yes | No | No | Yes | Yes | Yes | Yes | Yes | Yes | Unclear | 8 |
| Ivana et al.(2016) | Yes | Yes | No | No | Yes | Yes | Yes | Yes | Yes | Yes | Yes | 9 |
| Jolanta et al.(2005) | Yes | Yes | Yes | No | Yes | No | Yes | Yes | Yes | Yes | Unclear | 8 |
| Loai et al.(2017) | Yes | Yes | No | Yes | Yes | No | Yes | Yes | Yes | Yes | Unclear | 8 |
| Mirjana et al.(2021) | Yes | Yes | Yes | No | Yes | Yes | Yes | Yes | Yes | Yes | Unclear | 9 |
| Neslihan et al.(2023) | Yes | Yes | Yes | Yes | Yes | Yes | Yes | Yes | Yes | Yes | Unclear | 10 |
| Nishna et al.(2024) | Yes | Yes | Yes | Unclear | Yes | Yes | Yes | Yes | Yes | Yes | Unclear | 9 |
| Rezvan et al.(2016) | Yes | Yes | Yes | No | Yes | Yes | Yes | Yes | Yes | Yes | Unclear | 9 |
| Sahba et al.(2019) | Yes | Yes | No | No | Yes | Yes | Yes | Yes | Yes | Yes | Unclear | 8 |
| Salehi et al.(2024) | Yes | Yes | Yes | Yes | Yes | Yes | Yes | Yes | Yes | Yes | Unclear | 10 |
| Siudikiene et al.(2006) | Yes | Yes | No | Yes | Yes | Yes | Yes | Yes | Yes | Yes | Unclear | 9 |
| Swanljung et al.(1992) | Yes | Yes | No | No | Yes | Yes | Yes | Yes | Yes | Yes | Unclear | 8 |

**Table 1: AHRQ Research Checklist (Cross-sectional).**

**Figure 1. Forest plot of the pooled effect size for dmft**

**Figure 2. Forest plot of the pooled effect size for DMFS**

**Figure 3. Sensitivity analysis of DMFT**

**Figure 4. Funnel plot for DMFT**

**Figure 5. Sensitivity analysis of dmft**

**Figure 6. Funnel plot for dmft**
