# Supplementary material for: Association between type 1 diabetes mellitus and dental caries in children and adolescents: a meta-analysis and Mendelian randomization study
Source: Front Endocrinol (Lausanne). 2026 Sep 7;17:1929819. doi: 10.3389/fendo.2026.1929819 (PMC13590302; doi:10.3389/fendo.2026.1929819)
Supplement: Supplementary file 2 [file SupplementaryFile2.docx]

**Figure 1. Scatter plot of single nucleotide polymorphisms associated with type 1 diabetes mellitus and permanent tooth caries**

**Figure 2. Leave-one-out analysis of single nucleotide polymorphisms for the association between type 1 diabetes mellitus and permanent tooth caries**

**Figure 3. Forest plot of single nucleotide polymorphisms associated with type 1 diabetes mellitus and permanent tooth caries**

**Figure 4. Funnel plot of the effect of genetically predicted type 1 diabetes mellitus on permanent tooth caries**

**Figure 5. Scatter plot of single nucleotide polymorphisms associated with type 1 diabetes mellitus and primary tooth caries**

**Figure 6. Leave-one-out analysis of single nucleotide polymorphisms for the association between type 1 diabetes mellitus and primary tooth caries**

**Figure 7. Forest plot of single nucleotide polymorphisms associated with type 1 diabetes mellitus and primary tooth caries**

**Figure 8. Funnel plot of the effect of genetically predicted type 1 diabetes mellitus on primary tooth caries**
